# Degenerative Cervical Myelopathy: Clinical Presentation, Assessment, and Natural History

**DOI:** 10.3390/jcm10163626

**Published:** 2021-08-17

**Authors:** Melissa Lannon, Edward Kachur

**Affiliations:** Division of Neurosurgery, McMaster University, Hamilton, ON L8S 4L8, Canada; melissa.lannon@medportal.ca

**Keywords:** degenerative cervical myelopathy, cervical spondylotic myelopathy, cervical decompression

## Abstract

Degenerative cervical myelopathy (DCM) is a leading cause of spinal cord injury and a major contributor to morbidity resulting from narrowing of the spinal canal due to osteoarthritic changes. This narrowing produces chronic spinal cord compression and neurologic disability with a variety of symptoms ranging from mild numbness in the upper extremities to quadriparesis and incontinence. Clinicians from all specialties should be familiar with the early signs and symptoms of this prevalent condition to prevent gradual neurologic compromise through surgical consultation, where appropriate. The purpose of this review is to familiarize medical practitioners with the pathophysiology, common presentations, diagnosis, and management (conservative and surgical) for DCM to develop informed discussions with patients and recognize those in need of early surgical referral to prevent severe neurologic deterioration.

## 1. Introduction

Degenerative cervical myelopathy (DCM) is now the leading cause of spinal cord injury [1,2], resulting in major disability and reduced quality of life. While precise prevalence is not well described, a 2017 Canadian study estimated a prevalence of 1120 per million [3]. 

DCM results from narrowing of the spinal canal due to osteoarthritic changes. This narrowing leads to chronic spinal cord compression and neurologic disability. Symptoms may range from mild dysfunction, including numbness or decreased dexterity in the upper extremities, to severe dysfunction including quadriparesis and incontinence. Importantly, clinicians should note that paresthesia in the extremities may be the first sign and is frequently overlooked by patients and providers due to its mild nature. This variable pattern of presenting symptoms may lead to a delay in diagnosis of up to 2 years [4].

Early diagnosis and surgical management may improve neurologic and overall outcomes for these patients and, importantly, prevent progressive deterioration.

## 2. Topics

### 2.1. Pathophysiology

Degenerative changes in the spine are considered a normal part of the aging process. The cervical spine is particularly prone to degenerative changes due to the mobility of this region. Typically, the degenerative process that culminates in DCM begins with deterioration of the intervertebral disk [5,6,7]. The intervertebral disk normally acts to distribute pressure evenly across vertebral endplates and facet joints. Normal aging leads to loss of proteoglycans and dehydration of disks, causing loss of elastic and supportive structure. As the disk collapses, it bulges posteriorly, narrowing the spinal canal and compressing the spinal cord at that level. Resultant decreased disk height produces shortening of the spinal column, ultimately producing abnormal spinal mechanics [1,7]. These altered mechanics further contribute to osteoarthritic and osteophytic changes that may worsen narrowing. 

In addition to changes related to disk degeneration, the ligamentum flavum can thicken and buckle anteriorly toward the spinal cord, also resulting in compression. Finally, the posterior longitudinal ligament may contribute to degenerative cervical myelopathy by direct compression of the cord in the event of ossification of the posterior longitudinal ligament (OPLL) [5,6,7].

As these changes occur, stiffening of affected structures may result. To compensate, adjacent segments of the spine may develop hypermobility, which may further contribute to instability and degeneration as the process progresses. 

With these mechanical changes, abnormal repetitive movement of the cervical spine may cause spinal cord irritation and compression. For example, flexion may compress the spinal cord against anterior osteophytes and intervertebral disks, while hyperextension may lead to compression between the posterior aspect of the vertebral bodies anteriorly and hypertrophied ligamentum flavum posteriorly [8]. 

The aforementioned compressive factors produce vascular changes within the cord, inducing ischemia and inflammation [5,7]. With these changes, chronic compression may lead to demyelination, astrogliosis, and axonal degeneration. Endothelial damage may promote further cellular injury through disruption of the blood–spinal cord barrier [5,7]. Ultimately, this histopathologic pattern leads to cell loss and the subsequent functional decline observed clinically in patients [5,7].

Interestingly, recent studies have shown an association between DCM and cerebral reorganization, seemingly to compensate for functional impairment. The majority of studies have focused on cortical reorganization, however similar changes have been observed in the brainstem [9] and the thalamus [10]. This reorganization has been seen across a number of modalities, including arterial spin labelling functional MRI (fMRI) [11], blood oxygen level dependent (BOLD) fMRI [10,12], and navigated transcranial magnetic stimulation (nTMS) [13].

Congenital cervical spinal stenosis, defined as a sagittal canal diameter less than 13 mm [14] or a Torg-Pavlov (canal diameter/vertebral body diameter) ratio less than 0.82 [15], is recognized for its significant role in predisposing patients to DCM [16,17,18]. Congenital narrowing of the canal produces a vulnerability in the spinal cord to even minor compression from factors described above. It has been suggested that the narrow canal seen in these patients reduces cerebrospinal fluid volume at stenosed levels, impairing the cushioning effect of kinetic energy in the setting of minor trauma and other dynamic injury mechanisms described here [19]. As such, these patients should be monitored regularly for early onset myelopathic symptoms. 

### 2.2. Presentation

No pathognomonic sign exists for DCM. Therefore, clinicians must be cognizant of the constellation of symptoms in this variable presentation. Initially, patients with DCM most commonly present with paresthesia in one or more extremities. Patients may also report decreased dexterity, often described as “clumsiness” with buttons and zippers or changes in penmanship. Patients may note changes in mobility or frequent falls. 

DCM carries a slow progressive course, so while paresthesia is commonly an early symptom, patients may present at any point along the disease course with any number of symptoms, including weakness, sensory change, decreased dexterity, and gait abnormality. Neck pain may or may not be present. Bowel and bladder symptoms may occur, however clinicians should keep in mind that these symptoms are rare and indicative of severe injury to the spinal cord [7].

Physical examination is an important aspect of diagnosis in DCM. Patients should be thoroughly assessed for weakness, particularly in the intrinsic muscles of the hands. Patients commonly exhibit hyperreflexia, clonus at the ankles and patellae, spasticity, and abnormal Babinski and Hoffmann signs, as well as loss of sensory proprioception [7].

A number of classification systems have been generated to assess severity of DCM. The most commonly utilized is the modified Japanese Orthopaedic Association (mJOA) classification, grading motor dysfunction in both upper and lower extremities as well as sensation and bladder control to characterize patients as mild (mJOA 15–17), moderate (12–14), or severe (0–11) (Table 1) [20].

Numerous other classification scales have been utilized in the literature, including the Myelopathy Disability Index, Prolo Scale, and Nurick Scale [22,23,24,25,26,27].

The Nurick Grading Scale focuses primarily on gait assessment, ranging from grade 0 (signs and symptoms of root involvement without evidence of spinal cord disease) to 5 (chairbound or bedridden) [26]. Although commonly utilized and frequently correlated with surgical outcome, the Nurick score is considered less sensitive than the mJOA given its focus on lower limb function. One systematic review was unable to find a conclusive association with a number of predictors of outcome for DCM, unlike the more widely utilized mJOA score [28].

The Neck Disability Index (NDI) is a ten item self-assessment measure developed to assess disability in patients with neck pain following “whiplash” injury [29]. It is now widely utilized in the evaluation of operative spine patients. The domains assessed in the NDI include pain intensity, personal care, lifting, reading, headache, concentration, work, driving, sleep, and recreation. The challenge in adapting the NDI to DCM patients is that function, not pain, is the primary concern [30].

El-Zuway et al. suggested that these myelopathic scales are inherently subjective in nature. As a result, they proposed a ten-point myelopathic scale for DCM based on myelopathic signs from clinical examination. Statistically, this scale significantly correlated with postoperative improvement in DCM patients, but was based on a small number of patients (*n* = 36) and further studies are needed to validate this scale [31].

Each of the proposed scales provides another aspect of assessment and means to follow patients both pre and postoperatively. However, in general, it is believed that DCM is reasonably well followed with the mJOA in conjunction with objective testing of DCM patients with examination of myelopathic signs and objective measures of grip strength, dexterity, balance, and gait [31,32]. As such, most recommendations for determining severity of DCM in patients and clinical decision making primarily utilize mJOA. 

### 2.3. Differential Diagnoses

A number of differential diagnoses may present similarly to DCM. These conditions may be differentiated through comprehensive assessment. In addition, one systematic review identified MRI as the most valuable investigative tool to differentiate DCM from other clinical entities [33]. See Table 2.

### 2.4. Diagnosis

Thorough neurologic examination is the first step in diagnosis of DCM, followed by magnetic resonance imaging (MRI) to assess for spinal cord compression and confirm the diagnosis. It is important to consider that degenerative changes are common in asymptomatic patients, with 98% of healthy patients in their 20s showing degenerative disk disease on MRI [34]. As such, MRI findings should be carefully interpreted in the context of clinical signs and symptoms. 

The absence of a cerebrospinal fluid (CSF) signal on T2-weighted images (T2WI) allows for assessment of cord compression, while cord signal change has been associated with disease severity in cervical myelopathy. Specifically, T1-weighted imaging (T1WI) hypointensity has been noted as particularly important and indicative of cord injury associated with more severe functional impairment, higher frequency of myelopathic findings, and decreased potential for recovery [35].

**Figure 1:**
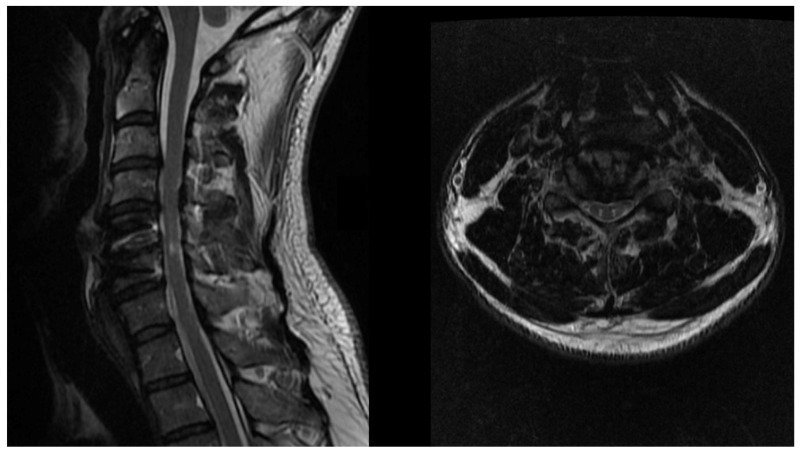
‘Snake eyes appearance’ sign on MRI. Bilateral, circular symmetric foci can be observed within the grey matter on T2WI MRI, thought to represent cystic necrosis secondary to chronic compression and vascular insufficiency.

Another sign on MRI that has been associated with cervical myelopathy is the “snake eyes appearance” sign, whereby bilateral, symmetric, hyperintense circular foci are seen within the gray matter of the spinal cord on T2WI (Figure 1). This finding is thought to represent cystic necrosis at the junction of the central gray matter and the posterior ventrolateral column, in addition to cell loss in the anterior horn. Chronic mechanical compression and vascular insufficiency are thought to be the most significant contributors to this pathogenic process. Although the literature is sporadic and inconsistent, this finding has been associated with negative prognosis for recovery in nearly half of patients in whom it is identified [36]. 

Martin et al. suggested that the primary purpose of MRI in DCM is to establish the diagnosis and for surgical planning. In their longitudinal study of DCM patients, MRI had a sensitivity of only 28% in detecting clinical deterioration of DCM and should not be relied upon as a measure to follow DCM patients [32].

Plain radiographs with flexion and extension views may be beneficial in these patients to rule out instability and assess the need for surgical instrumentation in planning, but for diagnostic purposes, clinical examination and MRI remain the mainstay. 

Computed tomography (CT) of the cervical spine may be useful for surgical planning to detect the degree of degenerative changes, osteophytes, and for instrumentation planning. CT myelogram may be utilized in rare instances to provide information on spinal cord compression in patients with contraindication to MRI or in cases where excess artifact exists due to previous instrumentation. 

Advanced imaging techniques have allowed improved investigation of microstructural and functional changes within the spinal cord as a result of DCM. In the future, these tools may provide improved diagnoses and prognostication for patients. In particular, diffusion tensor imaging (DTI) may be useful in identifying patients likely to benefit from surgical intervention. This technique uses directional diffusivity of water in each voxel to measure axonal integrity. The most reliable measure in DTI studies with DCM patients is fractional anisotropy (FA), measured from 0 (isotropic diffusion—same in all directions) to 1 (anisotropic—all in one direction). One systematic review found preoperative FA at the level of most severe spinal cord compression correlated closely with mJOA scores and postoperative mJOA changes [37]. It may also allow earlier detection of spinal cord injury in DCM [38].

Other advanced MRI techniques used in recent DCM literature include magnetization transfer (MT), myelin water fraction (MWF), and MR spectroscopy [35]. An ongoing study is investigating the role of microdiffusion imaging (MIDI) in DCM. This modality utilizes diffusion-weighted imaging (DWI) postprocessing to detect tissue alterations in each voxel [39]. 

Further adjuncts to diagnosis may be used, particularly in complex cases with multiple comorbidities with potential to cloud the clinical picture (e.g., patients with peripheral neuropathy or a previous peripheral nerve injury). In these cases, electromyography (EMG), electroneurography, and evoked potentials may be beneficial. Compared with healthy subjects, a number of surface EMG changes have been observed in DCM patients. Of these, prolonged duration activation of tibialis anterior was particularly useful clinically and a lack of coactivation of gastrocnemius suggested the presence in this finding may be due to impaired proprioception in DCM [40,41]. A number of studies have utilized somatosensory evoked potentials (SEPs) to illustrate dorsal column dysfunction in 24–100% of patients, depending on nerve distribution tested (lower limb, ulnar nerve, median nerve) [42,43,44,45]. However, upper limb SEPs were of no utility in patients without sensory changes and lower limb SEPs cannot provide information regarding localization. Motor evoked potentials (MEPs) have also been frequently utilized and most consistently demonstrate a prolonged central motor conduction time, with abnormalities in distal upper extremities for most DCM patients [40,46,47,48,49].

**Figure 2:**
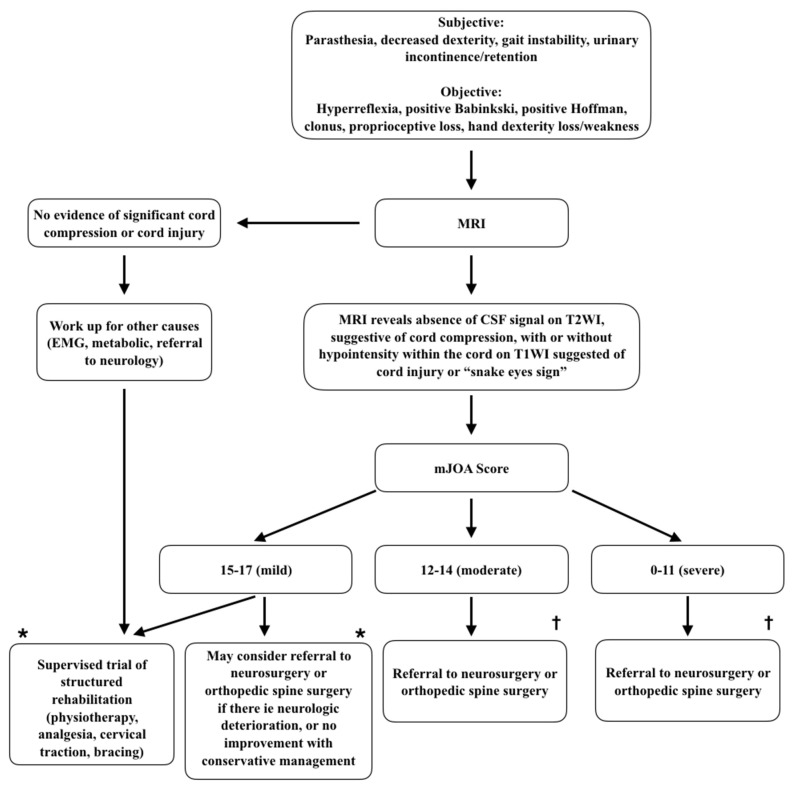
Approach to degenerative cervical myelopathy, summary of diagnostic and management (adapted from [50]). This summary decision tree can be used to guide decision making for medical practitioners. * denotes very low quality evidence with weak recommendation. † denotes moderate quality evidence with strong recommendation. This summary is based on Fehlings M, et al. [50].

Although the sensitivity of these modalities is considered quite high, they lack specificity and are ineffective in determining disease severity. Tools such as SEPs and MEPs are most useful in ruling out peripheral neuropathies and other differential diagnoses in complicated patients. While there is a diagnostic role for a number of modalities in DCM, the gold standard at this time remains thorough clinical assessment and MRI (Figure 2).

### 2.5. Natural History and Conservative Management

The first description of the natural history of degenerative cervical myelopathy was provided by Lees and Turner, who followed 44 patients with clinical myelopathy at St. Bartholomew’s Hospital in London. They observed a variety of durations of exacerbation, with long periods of latency interspersed. The authors noted it was common for patients to progressively decline with each exacerbation. Many patients were followed beyond five years (one patient up to 40 years) and at last follow up, 4.5% of patients had no disability, 6.8% were reported as mild disability, 47.7% moderate disability, and 40.9% of patients had severe disability. In spite of these poor outcomes, investigators concluded that a “very conservative approach” should be taken to degenerative cervical myelopathy [51].

More recent literature suggests 20–62% of patients with degenerative cervical myelopathy will have progressive neurologic deterioration within six months [52]. One randomized controlled trial compared patients undergoing anterior decompression (*n* = 22), corpectomy (*n* = 6), or laminoplasty (*n* = 5) with conservative measures including cervical collar, anti-inflammatory medications, bedrest, and avoidance of high-risk activities (e.g., heavy lifting, slippery surfaces, manipulation therapy, or prolonged neck flexion). There was no significant difference between groups in mean mJOA scores over a three year period nor the ten year period [53].

By contrast, one randomized controlled trial included functional assessments whereby blinded observers rated, by video recording, the ability of patients to perform activities of daily living, including buttoning shirts, brushing hair and teeth, putting on shoes, walking, running, and going up and down stairs. Of those patients conservatively managed, the number with declining scores increased over the course of follow-up from 6.3% at one year to 27.3% at three years. This change was not observed in operative patients, where the scores remained stable over time [54]. 

Similarly, a prospective study compared surgical treatment with conservative therapy including analgesia, physiotherapy, bedrest, cervical traction, and bracing. At mean follow-up at 29.8 months, surgical patients exhibited significant improvements in overall function, work, and social activities compared with conservatively treated counterparts [55].

A recent study by Martin et al. investigated the functional outcome in DCM patients treated nonoperatively in an ambispective longitudinal study. Deterioration of mJOA scores over a mean 30.3 months was observed in patients with a new diagnosis of DCM (57%, *n* = 95) and of recurrent DCM diagnosed at another level following surgery for DCM at the alternative level (73%, *n* = 22). The deterioration occurred with mild, moderate, and severe cases of DCM. The authors concluded that DCM appears to have a poor natural history and serial assessment by a battery of tests assessing for grip strength, dexterity, balance, and gait, in addition to the mJOA, in order to detect clinical deterioration where surgery would be indicated [32].

The wide variability in rate of deterioration may be related to the various methods of assessment in DCM. Further prospective studies are needed to better delineate the natural history of DCM. 

One notable risk for patients with cervical myelopathy is the development of myelopathic symptoms secondary to minor trauma, particularly with neck hyperextension. There is a paucity of literature assessing the true prevalence of spinal cord injury from minor trauma in these patients, but it remains a concern for care providers nonetheless.

### 2.6. Surgical Management

The decision to proceed with surgery for DCM requires a comprehensive discussion between the patient and medical and surgical providers. It should be clear from the start that the objective of surgery is to prevent further neurologic deterioration, as returning the patient to baseline is sometimes an unattainable outcome. However, literature does support the possibility of improvement, with one large retrospective, multicenter study of 2156 patients showing significant improvement in 18.8% of patients (2-point improvement in mJOA scores) between baseline and 3 month follow-up, with continued improvement to 12 month follow-up in patients with severe baseline scores [56]. 

As described previously, indication for surgical intervention is symptomatic myelopathy, especially if progressive, in conjunction with radiologic confirmation of cord compression and exclusion of concomitant contributing pathologies [23,57].

A number of surgical approaches exist for DCM and can be performed via anterior or posterior approaches, with or without the need for spinal fusion. Anterior decompression requires removal of the intervertebral disk (diskectomy), with contemporary approaches, including anterior fusion to prevent late disk space collapse and subsequent failure with recurrent symptoms, as had historically been the case. In some cases, the vertebral body is also removed (corpectomy) and the disk or vertebral body is replaced with an intervertebral cadaveric bone graft, iliac crest autograft, fibular allograft, or polyethereterketone or titanium cages. Anterior plating is often used adjunctively to provide further stabilization. Anterior approaches are often selected for patients with ventral compression, kyphosis, and/or compression at one to three levels [23,57]. In select cases with appropriate preoperative alignment, typically in younger patients, disk arthroplasty may be used as an alternative to anterior fusion [58].

For patients with multilevel disease, significant ligamentum flavum hypertrophy, or congenital narrowing of the canal posterior approaches are more favorable. These approaches are achieved through laminectomy (with or without fusion) or laminoplasty. Laminectomy involves removing the posterior elements (bilateral laminae, spinous processes, ligamentum flavum) to increase the diameter of the spinal canal. Commonly, lateral mass screws are placed with connecting rods bilaterally to provide stabilization and allow time for bone fusion to occur. In patients with loss of lordosis or evidence of instability and listhesis on preoperative radiographs, fusion is of particular importance [23,57]. Laminoplasty can be achieved in patients without evidence of instability and with preserved lordosis. This procedure expands the diameter of the canal through hinging open the laminae, displacing them laterally or posterolaterally. The laminae are fixed in this hinged position with graft, sutures, or plates [57,59]. For complex patients, a combination of anterior and posterior approaches may be used. 

Risks associated with surgical decompression for DCM must be discussed at length with the patient, including risk of permanent neurologic compromise, osteomyelitis, diskitis, meningitis, gait disturbance, quadriparesis, bowel or bladder dysfunction, C5 palsy, injury to the vertebral and/or carotid arteries resulting in stroke, cerebrospinal fluid leak, dislodgement of bone grafts and/or hardware, adjacent segment disease with need for further surgery, and anesthetic risk. Specific to anterior approaches, risk of injury to the trachea and/or esophagus, and recurrent laryngeal nerve palsy should also be discussed. Increasing patient age and comorbidities substantially increase surgical risk for patients. Again, it should be highlighted that the goal of surgery is to prevent further neurologic deterioration, but a risk of surgery is that there is no change in disease course or no improvement in symptoms [57]. 

Intraoperative neuromonitoring (somatosensory evoked potentials, transcranial motor evoked potentials) is frequently utilized in spinal surgery, with some uptake in both anterior and posterior decompressions for DCM. A recent systematic review reported that intraoperative monitoring may be a helpful tool in these surgical procedures given its high sensitivity and specificity for intraoperative neural damage detection. However, at this time evidence is limited, with no criteria for indications for its use [60]. An earlier systematic review made similar conclusions, noting that MEP/SEP monitoring may provide a sensitive tool for detecting neurologic injury during anterior approaches, intraoperative changes are not specific, and its recognition has not been found to prevent neurologic injury or result in improved outcome [61]. With appropriate patient selection after thorough assessment, it should be noted that these approaches are commonly performed and risk of progression of DCM should be balanced with limited surgical risk in consideration of these patients. 

## 3. Conclusions

DCM is a common clinical entity with increasing prevalence. Patients with clinically progressive myelopathic symptoms and correlating radiographic evidence of cord compression should be referred for surgical evaluation if it is within the patient’s care goals to prevent further neurologic deterioration. Discussion regarding conservative management and role for surgery in the medical setting may occur, with referral to surgical expertise where appropriate.

## Figures and Tables

**Table 1:** The modified Japanese Orthopaedic Association (mJOA) Score (adapted from [21]).

Category	Score	Description
Upper Extremity Motor	0	Unable to move hands
	1	Unable to eat with spoon but able to move hands
	2	Unable to button shirt but able to eat with spoon
	3	Able to button shirt with great difficulty
	4	Able to button shirt with mild difficulty OR other mild fine motor dysfunction (marked change in handwriting, frequent dropping of objects, difficulty clasping jewelry, etc.)
	5	Normal hand coordination
Lower Extremity Motor/Sensation	0	Complete loss of movement and sensation
	1	Complete loss of movement, some sensation present
	2	Unable to walk but some movement
	3	Able to walk on flat ground with walking aid
	4	Able to walk without walking aid, must hold handrail on stairs
	5	Moderate to severe gait imbalance but able to take stairs without handrail
	6	Mild imbalance standing OR walking
	7	Normal walking
Upper Extremity Sensory	0	Complete loss of hand sensation
	1	Severe loss of hand sensation OR pain
	2	Mild loss of hand sensation
	3	Normal hand sensation
Urinary function	0	Inability to voluntarily urinate (requiring catheterization)
	1	Frequent urinary incontinence (more than once monthly)
	2	Urinary urgency OR occasional stress incontinence (less than once monthly)
	3	Normal urinary function

The mJOA is a 17 point score of functional disability specific to cervical myelopathy that includes upper extremity motor, lower extremity motor/sensory, upper extremity sensory, and urinary function components. This version has been slightly modified from one previously published by Tetreault L, et al. [21].

**Table 2:** Approach to differential diagnoses of DCM.

Differential Diagnosis	Differentiating Findings
Amyotrophic lateral sclerosis [7,33]	Presence of cranial nerve findings (e.g., dysphagia, dysarthria) Absence of sensory findings
Brain neoplasm	Presence of cranial nerve findings Lateralizing findings (e.g., unilateral weakness/sensory changes) Headache Vomiting Altered level of consciousness
Multiple sclerosis [7,33]	Visual changes Cranial nerve findings Fatigue
Peripheral nerve entrapment (e.g., carpal tunnel syndrome, ulnar neuropathy) [33]	Absence of upper motor neuron findings
Normal pressure hydrocephalus	Cognitive disturbances Speech or swallowing difficulty
Vitamin B deficiency [7,33]	Fatigue Cognitive disturbances Glossitis Visual changes

A list of differential diagnoses for consideration in patients presenting with signs and symptoms of DCM.

## Data Availability

No additional data was utilized for the creation of this manuscript.
